# Trichotillomania with trichobezoar in 11-year-old girl - difficulties of recognizing the disorder and possible complications: case report

**DOI:** 10.1192/j.eurpsy.2023.1495

**Published:** 2023-07-19

**Authors:** A. Klobučar, L. T. Dobrić, S. Drmić, Z. Mišak

**Affiliations:** 1 Department of Pediatrics, Department of Child and Adolescent Psychiatry, Children’s Hospital Zagreb; 2 Department of Psychiatry, Referral Centre for the Stress-related Disorders, University Hospital Dubrava; 3 Department of Pediatrics, Department of Pediatric Gastroenterology, Children’s Hospital Zagreb, Zagreb, Croatia

## Abstract

**Introduction:**

Trichobezoar is a rare entity that primarily occurs as a complication of psychiatric disorders, most often in adolescent and young females suffering from trichotillomania (TTM) and trichophagia. In many cases, children with TTM unwillingly admit hair pulling, deny ingesting hair and often feel ashamed of their disease and try to hide it.

**Objectives:**

Our main aim was to present an uncommon complication of TTM and trichophagia and to point out the importance of early diagnosis and prevention of complications of the disorder. Furthermore, we describe the role of a child’s psychological features and family dynamics in etiopathogenesis of TTM, as well as comorbidities and specific clinical presentation.

**Methods:**

Case report.

**Results:**

An 11-year-old girl was admitted to the pediatric department due to abdominal pain. After detailed pediatric differential diagnosis, trichobezoar was diagnosed and she was treated surgically. While she did not deny ingesting her hair, three months after surgery (TTM was dermatologically verified from the beginning of the treatment) she mentioned focused hair pulling for the first time. During individual cognitive behavioral psychotherapy the following was recognized in the patient: perfectionism traits, inhibition in expressing emotions, elements of depression, anxiety. During family psychotherapy elements of alexithymia were observed.

**Conclusions:**

Cooperation among medical experts (pediatrician, dermatologist, child psychiatrist, pediatric surgeon etc.) and awareness of this disorder is important for recognizing it at an early stage and starting the treatment, especially considering habit-forming mechanism, psychiatric comorbidity, emotional distress and preventing other complications including trichobezoars.

**Keywords:**

adolescents, trichobezoar, trichophagia, trichotillomania

**Disclosure of Interest:**

None Declared

